# Wireless Laser Range Finder System for Vertical Displacement Monitoring of Mega-Trusses during Construction

**DOI:** 10.3390/s130505796

**Published:** 2013-05-06

**Authors:** Hyo Seon Park, Sewook Son, Se Woon Choi, Yousok Kim

**Affiliations:** 1 Department of Architectural Engineering, Yonsei University, 134 Shinchon-dong, Seoul 110-732, Korea; E-Mail: hspark@yonsei.ac.kr; 2 Center for Structural Health Care Technology in Buildings, Yonsei University, 134 Shinchon-dong, Seoul 110-732, Korea; E-Mails: seuk2809@hanmail.net (S.S.); watercloud@yonsei.ac.kr (S.W.C.)

**Keywords:** laser displacement sensor, structural health monitoring, displacement measurement, irregular building, mega-truss, in-construction monitoring

## Abstract

As buildings become increasingly complex, construction monitoring using various sensors is urgently needed for both more systematic and accurate safety management and high-quality productivity in construction. In this study, a monitoring system that is composed of a laser displacement sensor (LDS) and a wireless sensor node was proposed and applied to an irregular building under construction. The subject building consists of large cross-sectional members, such as mega-columns, mega-trusses, and edge truss, which secured the large spaces. The mega-trusses and edge truss that support this large space are of the cantilever type. The vertical displacement occurring at the free end of these members was directly measured using an LDS. To validate the accuracy and reliability of the deflection data measured from the LDS, a total station was also employed as a sensor for comparison with the LDS. In addition, the numerical simulation result was compared with the deflection obtained from the LDS and total station. Based on these investigations, the proposed wireless displacement monitoring system was able to improve the construction quality by monitoring the real-time behavior of the structure, and the applicability of the proposed system to buildings under construction for the evaluation of structural safety was confirmed.

## 1. Introduction

Recently, the need for the aesthetics and commercial intent of buildings to coincide with the development of their construction technique has led to an increasing trend for high-rise and irregular buildings. This trend increases the difficulty of construction due to the design complexities inherent in increasingly detailed construction [1–3]. Furthermore, the management of the construction process must ensure high-quality construction to prevent safety accidents caused by substandard construction [4–7]. Therefore, construction monitoring using various sensors is urgently needed for both more systematic and accurate safety management and high-quality productivity in construction. In addition, there is growing interest in structural health monitoring (SHM) techniques and the establishment of real-time monitoring systems based on sensor technology [8–12].

In terms of the type of sensors employed in SHM, accelerometers are mainly employed in a traditional SHM system to identify changes in dynamic characteristics, such as natural frequency, mode shape, and modal damping, induced from damage in the structure [13]. However, it is difficult to perform a quantitative evaluation on the health condition and safety of structures based on the change of dynamic characteristics because these characteristics are easily affected by non-structural elements and environmental conditions. Furthermore, another confounding factor is that damage is typically a local phenomenon, which is captured by higher frequency modes. However, the vibration-based damage identification method, which generally relies on lower frequency modes, tends to capture the global response of the structure and is less sensitive to local structural changes [14,15]. For these reasons, strain-type sensors are adopted to directly measure the strain of a structural element [16–20]. The stress distribution estimated from a strain measurement can be utilized in the safety assessment of an element by comparing the stress distribution with the yield stress of the materials or the design strength of the structural members. Several types of strain gauges are used to monitor structural responses, including electrical strain gauges (ESGs), fiber optic sensors (FOSs), and vibrating wire strain gauges (VWSGs). The major issue with the data obtained from strain-type sensors is that the observed strain value represents a damage condition restricted to a small area of the member. Therefore, a large number of strain gauges are needed to improve the accuracy of a structural damage evaluation.

To overcome the aforementioned limitations involving acceleration and strain values, there have been efforts to directly measure the displacement of a structure using a global positioning system (GPS), a vision-based system, and laser Doppler vibrometers. The measured displacement data can be utilized as a damage index, from which the structural stability and quality of construction can be estimated. GPS represents a good alternative to a displacement measuring system [21–24]. However, its applicability is limited to flexible and high-rise building structures because its accuracy is limited to 1 cm in the horizontal direction and 2 cm in the vertical direction [25]. The other main limitation in the application of GPS is that this system is restricted to outdoor or open spaces because signals are received from satellites. Vision-based systems are also proposed to measure the displacement of a structure and demonstrated an acceptable accuracy in SHM [26,27]. However, the light requirement in a vision-based monitoring system makes it difficult to use such systems at night; although night vision technology exists, its use is not yet feasible in SHM. Laser Doppler vibrometers also perform very well, but, these instruments are not suitable for long-term monitoring systems [28]. In addition, laser displacement sensors have been employed for measuring the displacement of structures subjected various loads such as wind and earthquake load in previous researches [29–31]. However, these measurement systems were used in laboratory experiments where environmental conditions can be easily controlled, and therefore their applicability to the real structures has not been validated. Because the ultimate goal of SHM involves the continuous and automatic monitoring the structural behavior induced from various loadings, the stable performance of monitoring system applied in actual structures is important and urgently needed. In particular, long-term monitoring of deflection in elements resulting from sudden change of loading condition, which frequently occurs in construction process and is a crucial factor in the safety of structure, is very challenging task, and there have been limited studies conducted using the existing displacement monitoring system.

In this study, a wireless laser range finder system is employed to directly measure the deflection of structural members in an irregular building that is currently under construction. The monitored irregular building is composed of large cross-sectional members, such as mega-columns, mega-trusses, and edge truss, which secure the large spaces. The mega-trusses and edge truss that support this large space are of the cantilever type. Thus, any vertical displacement occurring at the free end of these members was set as a major monitoring target and directly measured using a laser displacement sensor (LDS). In addition, the salient feature of this monitoring system is that a manager can perform real-time monitoring automatically, and the data are sent to a sensor node and then transmitted to a remote host server via wireless communications with good applicability to the structure under construction. The wireless network employed in this measurement system also overcomes the weakness of existing wired monitoring systems, which require large budgets to build the cable network and are inconvenient to manage [32].

To validate the accuracy and reliability of the deflection data measured from the LDS, the vertical deflections of the mega-trusses and edge truss were also measured using a total station as a sensor for comparison with the LDS during the bent removal, which was thought to cause the largest deflection during this construction process. In addition, the deflection of the mega-truss upon bent removal was analyzed using a structural analysis program and was compared with the deflection measurements obtained from the LDS and total station. Finally, the solutions for several issues discovered through the application of such wireless monitoring systems using an LDS were proposed for this practical application while continuing to perform structural safety monitoring using the data measured using this sensor technology.

## 2. Displacement Monitoring System

**Figure 1.**
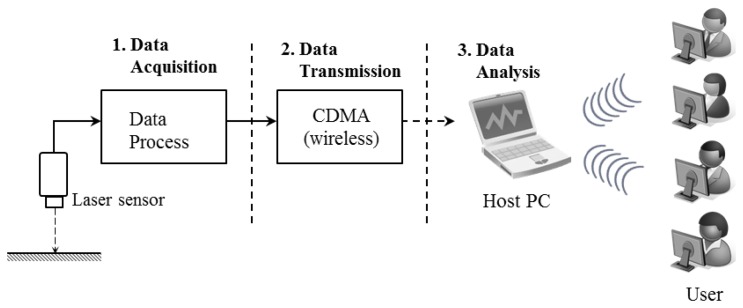
Data transmission process for the LDS.

A wireless measurement system was built to monitor the vertical deflections that occur in cantilever-type mega-trusses in an irregular building. The automatic wireless displacement measurement system consists of a sensor (LDS) and a sensor node. The sensor node is equipped with a battery, which supplies the power, and a code-division multiple-access (CDMA) driving circuit that delivers the data. The process for measuring data from the LDS can be divided into three steps, as illustrated in Figure 1.

### 2.1. Laser Displacement Sensor

The principle of non-contact laser sensors is largely divided into: (1) eddy-current, (2) optical, and (3) ultrasonic waves [33]. The eddy-current technique has a short measurement range and is therefore not suitable for long-distance measurements. The ultrasonic wave style has a long measurement range and can measure all objects; however, it is greatly affected by environmental factors, such as wind and temperature. Thus, when outside use is intended, the optical LDS technique is the most appropriate option.

In this research, an LDS was used to measure displacement in an economical and stable manner. An optical laser sensor (LLD-0100 model, JENOPTIK AG, Jena, Germany, Table 1) with a maximum sampling rate of 50 Hz was used in this study. This model has a built-in processor function such that the displacement data are outputted while power is supplied according to the preset data acquisition period. Thus, the distance between the LDS and object can be determined without a separate reader.

**Figure 2.**
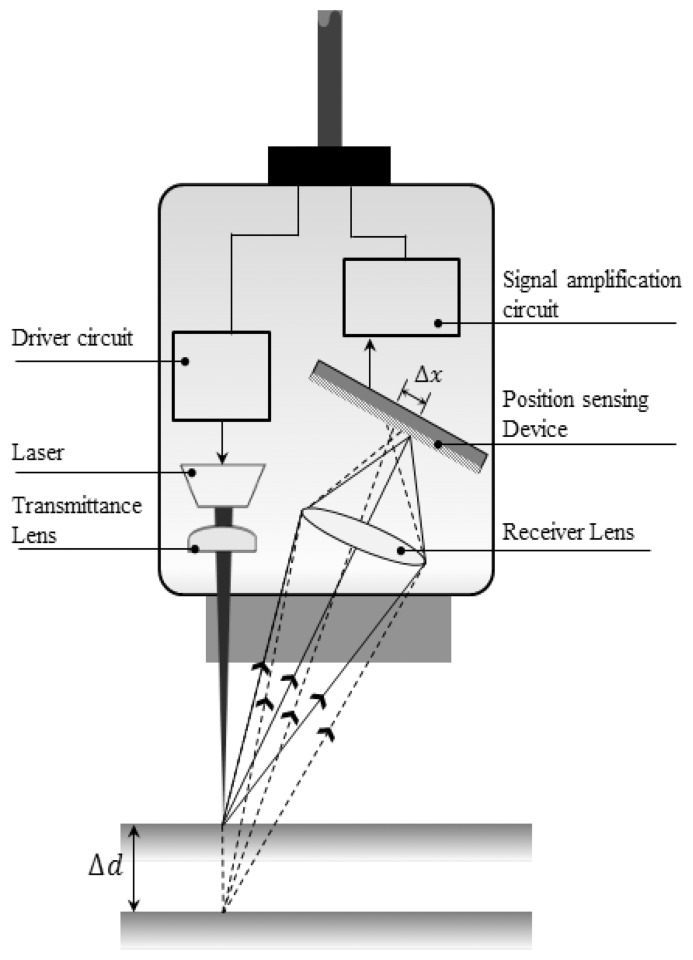
Principle of the LDS.

As depicted in Figure 2, the measurement principle of the optical LDS technique is that a laser beam, often with a diameter on the order of millimeters, is scattered when the target is reached, and this scattered beam creates an image on a one-dimensional position-sensing device that is then converted into an electrical signal. The distance between the LDS and target can be triangulated from the positional information of the imaged laser beam.

In a laboratory experiment, the environmental conditions can be easily controlled for the LDS application. However, when an LDS is used at a construction site, the measurement range, material of the object, and surrounding environment should be considered. First, a long reference distance and measurement range between the monitoring target and LDS are needed, considering the various obstacles that hinder stable measurement at a construction site. The measurement range of the LDS used in this research is 0.2–35 m, as indicated in Table 1, and can reach up to 150 m when reflecting plates are used. The accuracy of the LDS is 2 mm, which is relatively coarse. However, the measurement range is the primary requisite in this application, where the monitored structure is a large-scale irregular building structure during construction. High-accuracy LDSs with an accuracy on the order of micrometer are also available [34,35]; however, their short reference distance and measurement range which are less than 1 m are not applicable in this research. Therefore, a deflection accuracy on the order of millimeters is regarded as acceptable considering the trade-off between accuracy and the measurement range.

### 2.2. Wireless Monitoring System

The sensor node with the four-channel sensor interface can simultaneously receive data from four LDSs, which are connected through a cable for RS-422 communication. The remote wireless communication method (CDMA) [36] was used to send the data measured by the LDS from the construction site to the host personal computer (PC) in a remote location. The data sent to the host PC could be verified online in real-time via the integrated management software.

A continual power supply is important for the stable operation of automatic wireless monitoring systems. A cable layout plan is necessary for a direct power supply at a construction site, and the cables for such a power supply can reduce the applicability to a construction site from an operational and management perspective. Therefore, the wireless monitoring system developed in this research uses both a technique with low power consumption for the processor and a timer control to switch between the operating mode (data acquisition and transmission) and the sleep mode (power consumption minimized), which are synchronized with the data measurement periods to minimize power consumption. The processor is connected to a power source through the two circuits Regulator 1 and Regulator 2. Regulator 1 constantly supplies power to the processor so that it can operate with a minimum amount of power in sleep mode. Regulator 2 operates during the data measurement periods and activates the sensor node by supplying power to the sensor and CDMA drive circuit [37]. In other words, the lifespan of the battery was extended by minimizing the power consumed during times of inactivity rather than uniformly consuming power at all times. The power supply consisted of a rechargeable lithium-ion battery and was replaced approximately once every three months. The specifications for the sensor node are provided in Table 2.

## 3. Application to a Construction Site

### 3.1. Target Structure (D Building)

**Figure 3.**
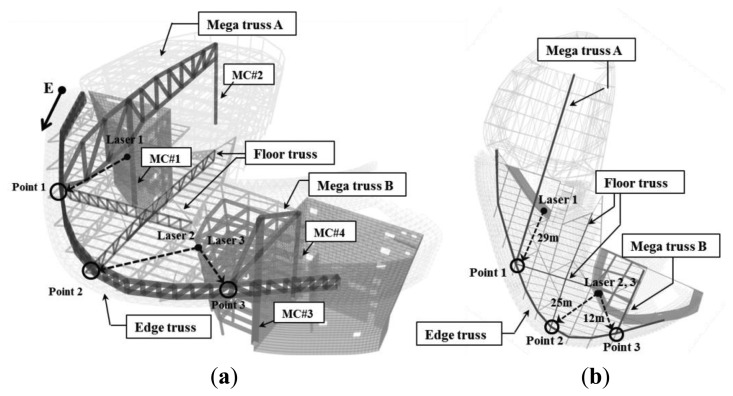
Target structure, D building. (**a**) Three-dimensional view of the main elements. (**b**) Cross-sectional plan of the main elements.

This research applied the proposed wireless displacement monitoring system to and conducted real-time monitoring of a building under construction (D building). The building is located in Seoul, the capital of South Korea; its construction began in April 2009 and is scheduled to be completed in 2013. As observed in Figure 3, the D building is an irregularly shaped building that will be used for various exhibitions as a museum, experience facility, convention hall, designed corporate offices and a sky lounge and consists of three underground floors and four stories.

**Figure 4.**
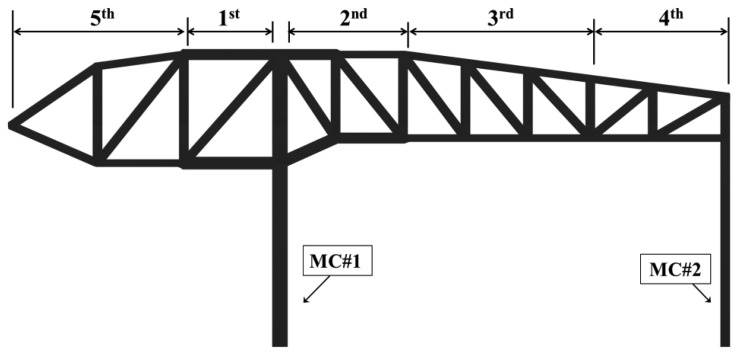
Mega-truss A.

**Figure 5.**
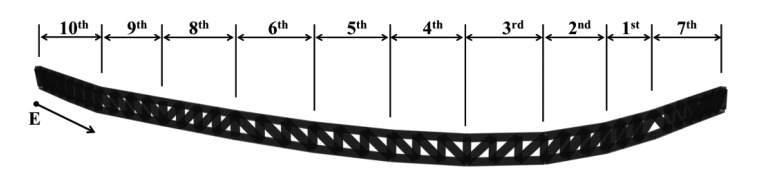
Edge truss.

The exhibition zone uses steel frames to present a large free space, and steel trusses with particularly large cross-sections were used to secure this space. These members can be grouped into mega-trusses A and B, an edge truss, and a floor truss according to their location. The composition of the complex space consists of four mega-columns (#1, 2, 3, and 4) that support mega-trusses A and B with additional smaller supports consisting of diverse steel numbers and cross-sectional sizes (Figure 3). As observed in Figure 4, mega-truss A was fabricated in several parts and constructed on-site through sequential erection and welding. The span length of the edge truss in Figure 5 is approximately 142 m; thus, it was divided into 10 parts for fabrication and similarly constructed via a sequential erection and welding process. To ensure structural safety while welding each part, a temporary bent was installed below the welding spots to support the weight of the structural member such as a mega-truss or edge truss.

In addition, each of the cores, which consist of mega-column #1 for one and mega-columns #3 and 4 for the other, is a reinforced-concrete (RC) structure, and these two cores are connected through the fourth floor slab used by the exhibition hall. As there is no column under the fourth floor slab, the dead load is sustained by the floor truss, which supports the center of this slab, and the edge truss supports its border and flows into the connected mega-truss, which is supported by the mega-column and RC core.

**Figure 6.**
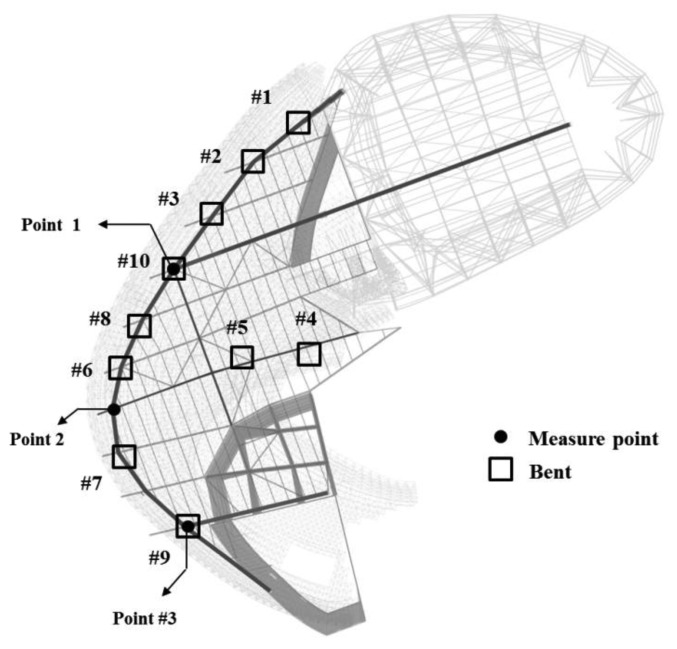
Removal schedule for temporary bents.

The distance from mega-columns #1 and #2 to the edge truss is 35 m and 12.3 m, respectively, and the mega-truss connected to the edge truss is of the cantilever type. The gravity load of the space frame, which is installed on the exterior panel, combines with that of the fourth floor slab, particularly during construction; thus, the deflection of the mega truss is a priority control and monitoring target during construction. In particular, the variations in the deflection of the edge truss were continually monitored as the 10 temporary bents that had been installed to support the construction of both the mega-truss and edge truss were individually removed following the order shown in Figure 6.

### 3.2. Measurement Setup

The vertical deflections of the edge truss were measured from three points (points 1, 2, and 3 in Figure 3). Point 1 is the connection point between mega-truss A and the edge truss, point 2 is the connection point between the edge truss and floor truss, and point 3 is the connection point between mega-truss B and the edge truss. The deflections that can occur at these three points due to the dead and live loads only were calculated using a commercial structural analysis program (MIDAS/GEN” ver.800, [38]), and the predicted camber of each point is listed in Table 3.

**Figure 7.**
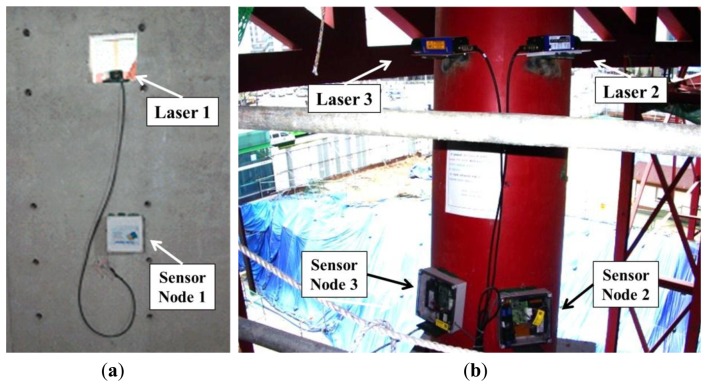
Installation location. (**a**) Laser sensor 1. (**b**) Laser sensors 2 and 3.

When measuring vertical deflection, it is best to measure the displacement by installing an LDS on the ground and reflecting it off of the target member. However, due to the conditions of the construction site, such as the various construction equipment and movements, and because the measurement range of the LDS is limited (maximum of 35 m), it was impossible to install these sensors on the ground. Therefore, in this research, the LDSs were installed horizontally within the building in the areas least affected by the construction to measure the data in a stable manner (Figure 7).

**Figure 8.**
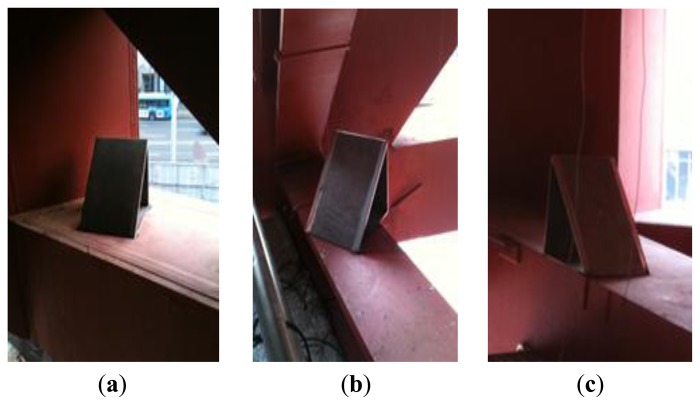
Triangle module installed at the measured point: (**a**) point 1, (**b**) point 2, and (**c**) point 3.

To determine the vertical displacement via the horizontal displacement measurements, a triangle module was welded to the target points, as illustrated in Figure 8. A module (300 × 300 × 300 mm) with a height (h) of 300 mm that forms a 45° angle to its base was welded onto the measurement point. The module was planned such that the horizontal displacement of the LDS and the measurement point would create a vertical deflection. In other words, because the triangle module was at 45°, determining its vertical displacement by measuring the horizontal displacement was possible using the principle that the distance (Δx) and vertical variation (Δz) were the same when the vertical deflection occurred in the z direction.

### 3.3. Total Station

To verify the accuracy and applicability of the deflections measured by the proposed wireless monitoring systems, the vertical deflections were also measured using a total station as a sensor for comparison with the LDS during the bent removal, which was thought to cause the largest deflection during this construction process.

**Figure 9.**
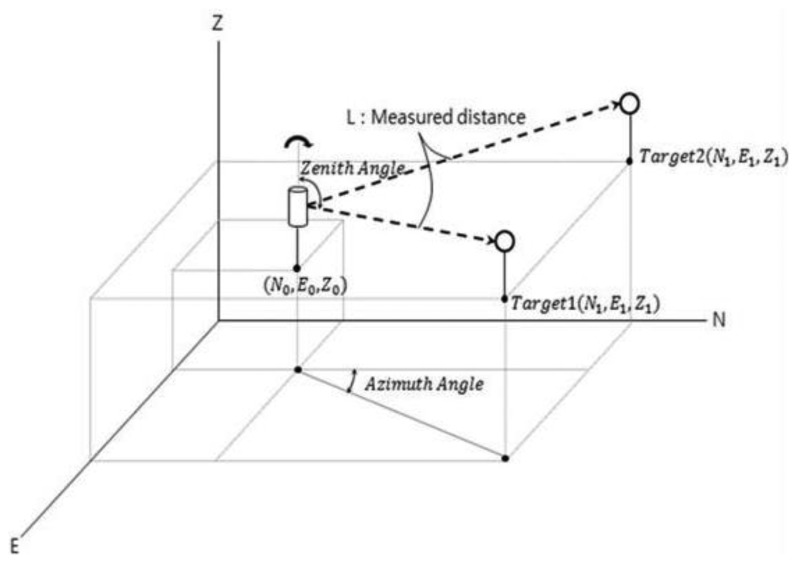
Measurement method of the total station.

**Figure 10.**
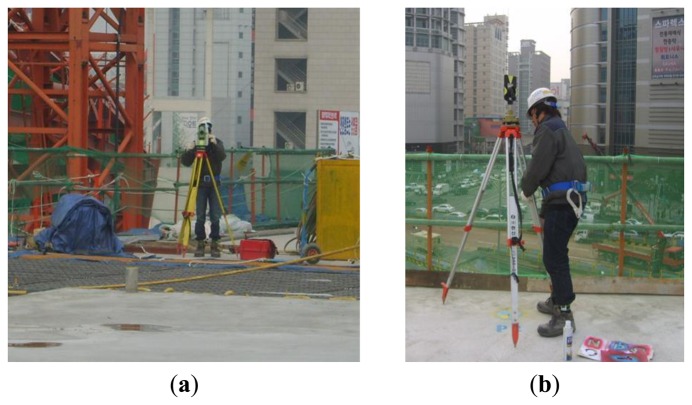
Total station measurement: (**a**) total station and (**b**) prism.

**Figure 11.**
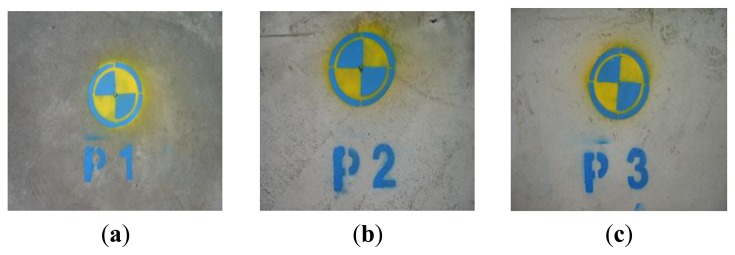
Prism: (**a**) point 1 mark, (**b**) point 2 mark, and (**c**) point 3 mark.

The total station combines an electro-optical instrument (electrical discharge machine (EDM)) to measure the angle with a theodolite to simultaneously measure distance. This device can determine the three-dimensional coordinates of the target point (Figure 9). The total station consists of four parts: a detector measuring the vertical angle of the up and down motions, a detector measuring the horizontal angle of the right and left motions, a distance gauge measuring the distance from the body to the target point, and a tilting sensor that measures and revises the level of the body. Prisms are installed at the target points, and a light wave with a known wavelength and frequency is transmitted (Figures 10 and 11). The distance can be determined from the time it takes the light wave to return, and the three-dimensional coordinates can be determined based on the azimuth and altitude. Therefore, the data measured using the total station are more accurate than those measured with the LDS, which only measures the one-dimensional distance. Thus, this research used the total station measurement as the real deflection when comparing and analyzing the results obtained using the LDS.

In this research, the prism was installed above the point where the LDS measured the deflection and only used the total station for measurements during the bent removal period. Figures 10 and 11 provide images of the installation of the total station at the construction site and the prism at the target point, respectively.

## 4. Evolution of Displacement during Construction

### 4.1. Evolution of Deflection at Each Point during Construction

After the welding connections of the fabricated mega-truss and edge truss were completed, the temporary bents were removed. The dead load of the structure that had been supported by the bents was then directly supported by these members, and the edge truss began to deflect. A total of 10 bends had supported the edge truss, and these bents were sequentially removed according to the construction schedule, as observed in Figure 6.

**Figure 12.**
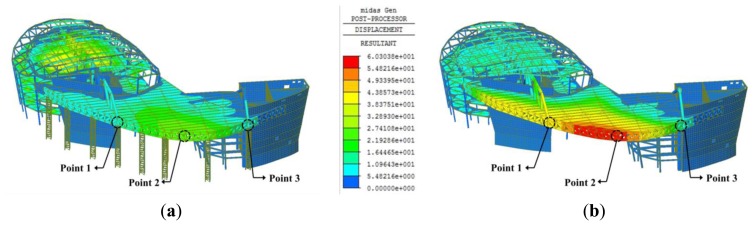
Calculated displacement of building: (**a**) before and (**b**) after bent removal.

The deflection of the edge truss upon bent removal was analyzed using a structural analysis program (MIDAS/GEN” ver.800, [38]) and compared with the deflection measurements obtained from the LDS and total station. When modeling the structure, the space frame, or external panel, was excluded to reflect the construction stage at the time of measurement, and the mega-trusses, edge truss, and floor trusses were modeled as rigid connections considering their large sectional size and welding construction. The analysis was performed by removing each temporary bent in accordance with the construction process, and the deflections at points 1, 2, and 3 for each step were analyzed. Figure 12 presents plots of the calculated vertical displacement of the structure both before and after the removal of the temporary bents. The maximum displacement occurs at point 2 on the edge truss after bent removal.

**Figure 13.**
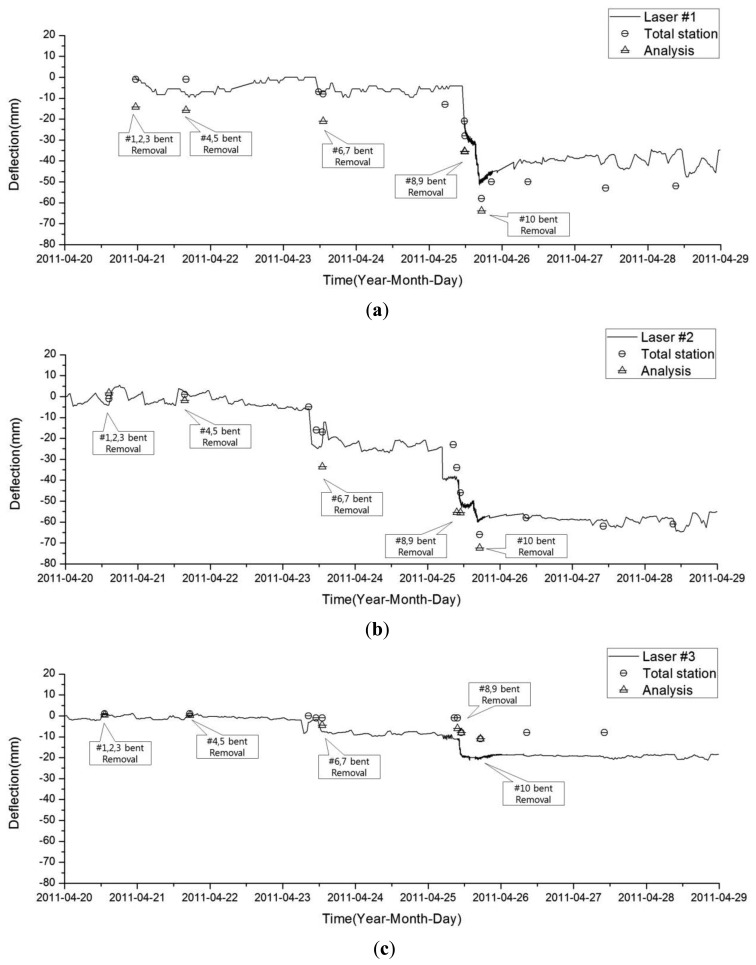
Variations of deflection during bent removal: (**a**) point 1, (**b**) point 2 and (**c**) point 3.

The plots in Figure 13 compare the values obtained from both the structural analysis and vertical deflection as measured by the LDS and total station for each measuring point. The LDS continuously measured each point every 30 min, and the total station was measured while each bent was being removed.

The difference in the deflection between the LDS and total station at point 2 was less than 5 mm, and changes occurred as the deflection increased with time (sequential removal of the bent). The vertical displacement determined for each point from the analysis overestimated the LDS and total station measurements. Nevertheless, the increase in sequential deflection due to bent removal was reproduced in the structural analysis results.

### 4.2. Comparison of the Deflection Values obtained from the LDS and Total Station

**Figure 14.**
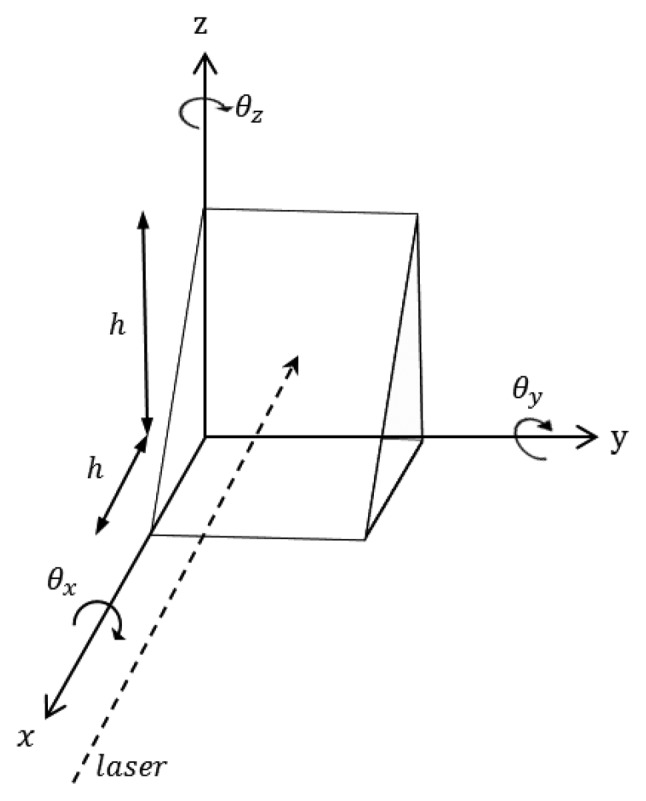
Variations in the measured value according to the rotation of the triangle module.

As demonstrated in Figure 13, the total station measurements were larger than those of the LDS at point 1; however, the results obtained at point 3 exhibited an opposite trend. The total station can measure the three-dimensional displacement of the target; however, the LDS can only provide data regarding the distance between the sensor and target point. This type of error generated in a pointer measuring system was previously investigated in pioneering research [39], and in this study, the discrepancy between two measuring systems is also investigated in the following manner. When structural deformation occurs, the triangular module on the target object also moves, and the error from the horizontal displacement and rotational components may be included in the measurement data with the absolute vertical displacement. In other words, the difference in distance caused by the rotation of the marked axis shown in Figure 14 can be obtained using Equations (1), (2), and (3). When the member and triangular module are attached to the x and y planes, Equation (1) provides the difference in distance between the LDS and the slope face of the module when the module rotates *θ* in the *θx* direction. Equations (2) and (3) provide the difference in distance between the LDS and module for the y and z axes, respectively:

(1) Aboutθx,Δx=h/2(sinθ+cosθ−1)

(2) Aboutθy,Δx=h{(1−cosθ)/(sinθ−cosθ)}

(3) Aboutθz,Δx=h/2{(1+sinθ−cosθ)/cosθ

**Figure 15.**
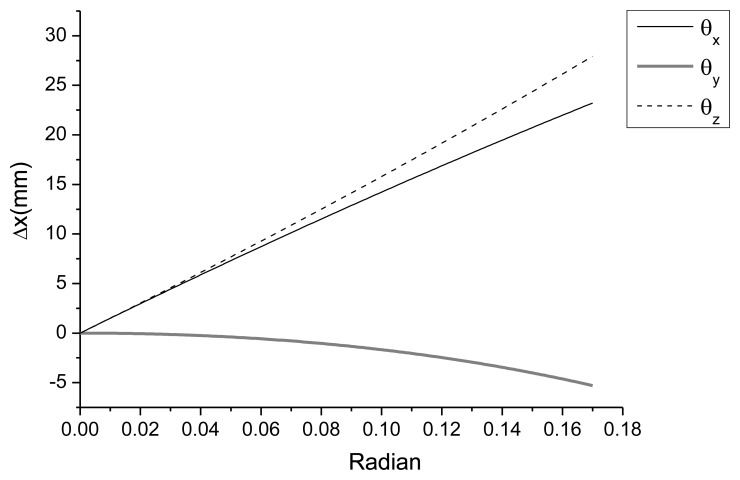
Change in horizontal distance due to rotation around each axis.

As demonstrated in Figure 15, the maximum distance between the LDS and target occurred when the module was rotated around the z axis. The minimum distance between the LDS and target occurred when the module was rotated around the y axis; however, this variation induced from the rotation around the y axis was small compared to the variations of the other axes.

As these results and the measurement data demonstrate, the largest vertical displacement occurred at point 2. Therefore, because the module at point 1 can be observed to rotate in the - *θx* direction, the horizontal distance between the LDS and target module decreases, and a value smaller than the real deflection observed from the total station is measured. In contrast, at point 3, the module rotates in the + *θx* direction, and the LDS measures a higher value than the real deflection. Therefore, the LDS measurement in Figure 13(a) is larger than that of the total station, whereas the LDS measurement in Figure 13(c) is smaller than that of the total station. That is, the effects of rotation are included in the results measured by the LDS in addition to the displacement by pure vertical deflection.

### 4.3. Detection of the Real-Time Behavior of Structures

April 25, 2011 was the last day of temporary bent removal, and bents 8, 9, and 10 were removed. The location of bent 9 was the connection point between mega-truss B and the edge truss (point 3). Bent 8 was the connection between mega-truss A and the edge truss (point 1). Thus, the locations of these bents have the largest effect on the LDS measurements. Furthermore, the deflection that occurs after removing all of the bents is caused by the dead load and can be used to estimate the safety of the construction.

**Figure 16.**
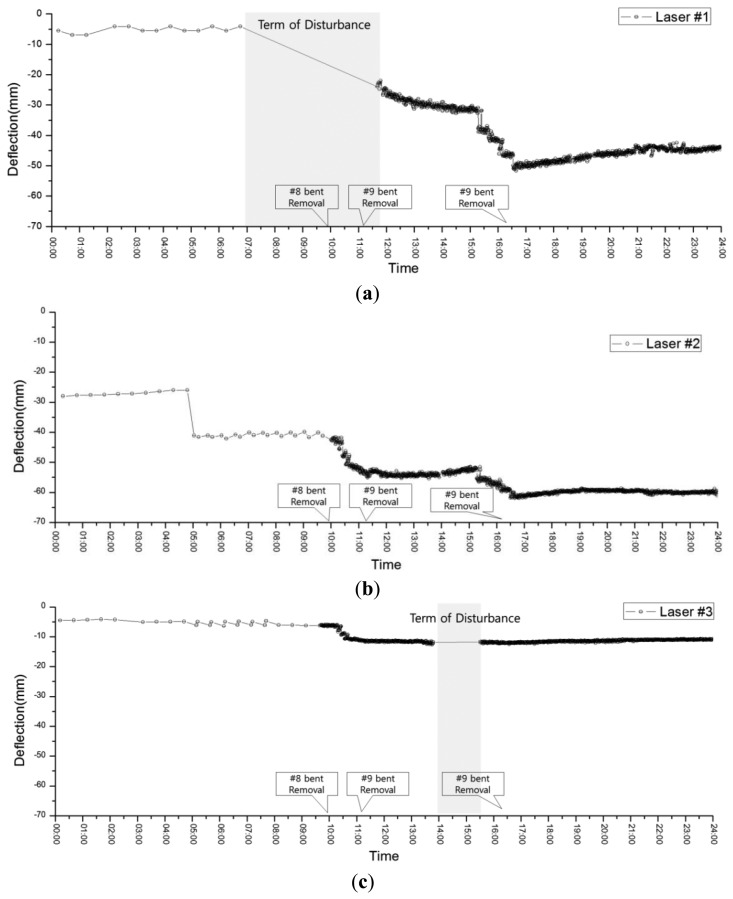
Measured deflection on April 25: (**a**) point 1, (**b**) point 2, and (**c**) point 3.

Figure 16 presents the measurement results at each point during the day of bent removal. At 10 AM, when the removal of bent 8 began, the LDS measurement interval was reduced from 30 min to 1 min to continuously acquire data from and to verify the deflection of each point in real time. However, a null-data period can be observed from 7 AM to 11 AM and from 2 AM to 3:30 PM at points 1 (Figure 16(a)) and 3 (Figure 16(c)), respectively. These null-data periods result from lost measurements due to the presence of obstacles, such as construction equipment, between the LDS and target during bent removal. After 5 PM, when all of the bents had been removed, stable measurements were obtained without large variations in the deflection. From the above results, it can be observed that the safety monitoring of the structures is possible, even when there are sudden changes to the load (e.g., bent removal in this study), via the flexible control of measurement intervals. Furthermore, the maximum deflection at each point after bent removal was smaller than the initial camber value (Table 3) determined from the predicted vertical deflections of 52, 67, and 13 mm at points 1, 2, and 3, respectively. Thus, the safety of the edge truss and mega-truss was confirmed.

## 5. Conclusions

This research proposed a displacement monitoring system using an LDS to perform real-time monitoring of an irregular large-scale building under construction, which consisted of structural members (namely, mega-trusses, mega-columns, and an edge truss) for large spaces. The boundary deflection of the broad slab, which was supported by a cantilever-type mega-truss and the edge truss, was targeted for the primary monitoring. The data measured by the LDS were sent to the sensor node, processed by a built-in processor, and finally transmitted via a CDMA method to a remote host PC server. This entire process was successfully automated. The following results were obtained from the deflection data measured from the proposed monitoring system

The deflection data measured from the LDSs accurately captured the cantilever behavior (*i.e*., deflection at the free end) caused by the removal of bents, which were temporarily installed at the free end of the mega-truss and edge truss during construction to prevent the deflection of the free end of the slab. Furthermore, the maximum deflection at each point after bent removal was smaller than the initial camber value determined from the predicted vertical deflections. Thus, the structural safety of the edge truss and mega-truss were confirmed.

From the deflection data collected from the total stations, which allowed for more accurate distance measurements, slight discrepancies between the two measurements were observed. The source of these permissible differences can be attributed to the movement of the triangular module on the target object, and the error from the horizontal displacement and rotational components might be included in the results measured by the LDS in addition to the pure vertical deflection. In the numerical simulation results, the increase in sequential deflection due to bent removal was also reproduced, although these results overestimated the LDS and total station measurements.

According to the above results, the proposed wireless displacement monitoring system based on the LDS was able to improve construction quality by monitoring the real-time behavior of the structure, and the applicability of the proposed system to buildings under construction for the evaluation of structural safety was also confirmed.

## Figures and Tables

**Table 1.** Specifications of the LDS.

**Model**	**Measurement Range (m)**	**Accuracy (mm)**	**Resolution (mm)**	**Temperature Range(°C)**	**Max. Sampling Rate**
LLD-0100	0.2–35	±2	0.1	−10–60	50 Hz

**Table 2.** Sensor node specification.

**Number of Channels**	**Power Consumption**	**Data Output**	**Software**
4	DC 7.4–24 V 100 mA (operation)	CDMA	LDSMS Client

**Table 3.** Camber of each measurement point.

**Location**	**Point 1**	**Point 2**	**Point 3**
Camber (DL + LL) (mm)	155.1	164.4	44.3
